# TLR4 modulates simvastatin’s impact on HDL cholesterol and glycemic control

**DOI:** 10.3389/fphar.2025.1655873

**Published:** 2026-01-16

**Authors:** Xiao Tian, Peixiang Zhang

**Affiliations:** Division of Endocrinology, Diabetes and Nutrition, Department of Medicine, University of Maryland School of Medicine, Baltimore, MD, United States

**Keywords:** dysglycemia, feeding-fasting cycle, HDL cholesterol, LPS–TLR4 signaling, PPARα, simvastatin

## Abstract

**Background:**

Statins reduce atherosclerotic cardiovascular risk by inhibiting HMG-CoA reductase and lowering LDL cholesterol, but their efficacy and adverse effects—particularly statin-associated dysglycemia—are tightly coupled to sterol regulatory element-binding protein (SREBP)–regulated cholesterol biosynthesis. Emerging work indicates that feeding–fasting cycles, rather than intrinsic circadian clocks, are the dominant drivers of hepatic SREBP activity and *de novo* lipogenesis. In parallel, the “feeding state” is now recognized to include metabolic endotoxemia, whereby gut-derived lipopolysaccharide (LPS) enters the circulation and activates Toll-like receptor 4 (TLR4), impinging on SREBP, liver X receptor (LXR), and peroxisome proliferator-activated receptor-α (PPARα) signaling. How this LPS–TLR4 axis interacts with statin action to shape lipid and glucose metabolism remains unknown.

**Methods:**

We leveraged the natural diurnal rhythm of food intake in mice to test how simvastatin timing relative to fasting–feeding cycles and LPS–TLR4 signaling influences metabolic outcomes. Simvastatin was administered either during the fasting (rest) phase by oral gavage or during the active feeding phase via chow admixture. Comprehensive metabolic phenotyping was integrated with hepatic transcriptomics and biochemical assays to interrogate SREBP-2–mediated autophagy, LXR/SREBP-1c activity, and PPARα signaling. To define the contribution of metabolic endotoxemia, parallel studies were performed in Tlr4-deficient mice.

**Results:**

In wild-type mice, fasting-phase simvastatin activated SREBP-2–dependent autophagy, augmented PPARα signaling, and increased HDL cholesterol but impaired glucose homeostasis. In contrast, feeding-phase simvastatin lowered HDL cholesterol while improving glucose tolerance and insulin sensitivity. Mechanistically, feeding elicited a surge in circulating LPS that suppressed hepatic oxysterol production, sensitizing the liver to further simvastatin-mediated oxysterol depletion and attenuation of LXR/SREBP-1c activity, thereby shifting the LXR/SREBP-1c/PPARα axis toward reduced HDL biogenesis and enhanced glycemic control. TLR4 deficiency abolished these feeding-phase effects, reversing the HDL-lowering and glucose-improving actions of simvastatin.

**Conclusion:**

The timing of simvastatin administration relative to feeding versus fasting exerts opposing effects on HDL and glucose metabolism, and these divergent outcomes are critically gated by feeding-induced LPS–TLR4 signaling. Aligning statin therapy with nutritional state, and potentially targeting the LPS–TLR4–SREBP/LXR/PPARα axis, may offer a tractable strategy to optimize lipid-lowering efficacy while mitigating dysglycemic risk.

## 1 Introduction

Atherosclerotic cardiovascular disease (ASCVD) remains the leading cause of mortality worldwide. By inhibiting the rate-limiting enzyme 3-hydroxy-3-methylglutaryl–coenzyme A (HMG-CoA) reductase in cholesterol biosynthesis, statins effectively lower low-density lipoprotein cholesterol (LDL-C) and reduce ASCVD events (Strandberg et al., 2024). Large randomized controlled trials and meta-analyses consistently show that a 1 mmol/L reduction in LDL-C yields approximately a 25% reduction in myocardial infarction and ischemic stroke (Cholesterol Treatment Trialists et al., 2010; Cholesterol Treatment Trialists et al., 2015). In addition to lowering LDL-C, statins can modestly raise high-density lipoprotein cholesterol (HDL-C) and exert pleiotropic benefits on endothelial function, oxidative stress, and inflammation (Zimmermann et al., 2020).

Despite this favorable cardiovascular profile, accumulating evidence indicates that statin therapy is associated with a modest but significant increase in the risk of new-onset diabetes, particularly at higher doses and among individuals with pre-existing metabolic risk factors. Recent meta-analyses have estimated a 10%–36% relative increase in incident diabetes with statin use, with intensive-dose regimens conferring greater risk than moderate-dose therapy (Cholesterol Treatment Trialists’ Collaboration, 2024). Observational studies and *post hoc* analyses of trials such as JUPITER and WOSCOPS have likewise reported higher fasting glucose, hemoglobin A1c, and diabetes incidence among statin-treated patients, especially in those with metabolic syndrome or impaired fasting glucose at baseline (Laakso and Fernandes Silva, 2023). Experimental studies have suggested multiple mechanisms by which statins may perturb glucose homeostasis, including impaired pancreatic β-cell function, reduced GLUT4-mediated glucose uptake in adipose tissue and skeletal muscle, and alterations in hepatic gluconeogenesis and glycogen metabolism through depletion of isoprenoids and disruption of small GTPase signaling. Yet other reports describe improved insulin sensitivity under specific conditions, underscoring the context-dependent nature of statin effects on glucose metabolism (Cholesterol Treatment Trialists’ Collaboration, 2024).

Because short–half-life statins such as simvastatin, fluvastatin, and lovastatin are rapidly cleared, their pharmacodynamic effects are highly sensitive to dosing time. In mice, oral administration of simvastatin produces a sharp plasma peak within 1 h, with concentrations falling back to near baseline by 12 h post-dose, mirroring its rapid clearance in humans (Fahmy, 2016). Accordingly, the timing of statin administration relative to circadian and metabolic cycles—particularly sleep–wake and feeding–fasting rhythms—has long been recognized as a critical determinant of lipid-lowering efficacy (Awad and Banach, 2018; Awad et al., 2017; Kim et al., 2013; Martin et al., 2002; Wang et al., 2023; Whitfield et al., 2000). A meta-analysis showed greater LDL-C reductions with evening dosing of short–half-life statins, consistent with peak nocturnal hepatic cholesterol synthesis (Awad et al., 2017). However, a Cochrane review reported no clinically meaningful differences between morning and evening dosing (Izquierdo-Palomares et al., 2016). suggesting that important modifiers of statin chronopharmacology remain unaccounted for. Among these, the feeding status at the time of statin intake—which directly influences hepatic sterol regulatory element-binding protein (SREBP) activation and lipid metabolism—has emerged as a key but underappreciated determinant of statin efficacy and metabolic side effects, including dysglycemia.

Recent human and animal studies indicate that feeding–fasting cycles, rather than intrinsic circadian clocks, are the dominant drivers of hepatic lipid and cholesterol metabolism (Greco et al., 2021; Stokkan et al., 2001; Vollmers et al., 2009). SREBPs are central to this regulation, controlling genes involved in cholesterol and fatty acid synthesis. Carbohydrate-rich, low-fat meals robustly activate nuclear SREBPs and their downstream targets, including HMG-CoA reductase, thereby enhancing *de novo* cholesterol synthesis, whereas fasting markedly suppresses nuclear SREBP-1 and SREBP-2 and their target genes, with refeeding rapidly restoring their activity (Horton et al., 2002). Feeding-driven oscillations in SREBP activity and cholesterol synthesis persist in clock-deficient models, underscoring the predominance of nutritional cues over circadian pacemaker signals (Greco et al., 2021; Stokkan et al., 2001; Vollmers et al., 2009). In line with this, the feeding–fasting cycle coordinately modulates SREBP activity, hepatic fatty acid and cholesterol synthesis genes (e.g., fatty acid synthase, HMG-CoA reductase), and liver cholesterol production rates (Greco et al., 2021; Horton et al., 2002; Kelley and Story, 1985; Stokkan et al., 2001; Vollmers et al., 2009); feeding can increase hepatic HMG-CoA reductase activity more than fourfold, paralleling surges in cholesterol synthesis. Plasma mevalonate, a downstream product of HMG-CoA reductase, shows a delayed peak ∼12 h after feeding in both rodents and humans, largely because of renal handling and nocturnal reductions in urinary output (Nakamoto et al., 2021). This temporal lag limits plasma mevalonate as a real-time surrogate of hepatic cholesterol synthesis (Cholesterol Treatment Trialists et al., 2010; Nakamoto et al., 2021). Nevertheless, the complete loss of mevalonate rhythmicity under prolonged fasting further underscores the dominant influence of feeding cues over intrinsic circadian regulation (Kelley and Story, 1985; Parker et al., 1984).

In keeping with the notion that nutritional context modifies statin action, clinical pharmacology studies have highlighted several food–statin interactions that alter statin pharmacokinetics and toxicity. Grapefruit juice, by inhibiting intestinal CYP3A4 and uptake transporters, markedly increases the area under the curve and peak concentrations of simvastatin and other lipophilic statins, and has been linked to myopathy and rhabdomyolysis in pharmacokinetic studies and observational cohorts (Bailey et al., 2013; Lilja et al., 1998; Neuvonen et al., 2006). High-fat meals can also influence the absorption and first-pass metabolism of some statins, leading to measurable changes in C_max and overall exposure in controlled studies (Lennernas, 2003). Surveys of ambulatory patients with dyslipidemia indicate that a substantial minority regularly combine statins with grapefruit products or high-fat evening meals, creating real-world opportunities for variable statin exposure and adverse events (Bailey et al., 2013). These clinical observations reinforce that food intake can meaningfully shape statin pharmacology, yet they have largely focused on specific food–drug interactions rather than the physiological feeding–fasting cycle itself. Thus, it remains unclear how feeding behavior as a whole shapes the integrated effects of statins on both lipid and glucose metabolism.

Importantly, compared with the fasting phase, the physiological “feeding state” encompasses not only postprandial insulin and nutrient fluxes but also gut–liver crosstalk mediated by microbial products. Meal ingestion promotes transient translocation of gut-derived lipopolysaccharide (LPS) into the portal circulation—a phenomenon termed metabolic endotoxemia (Cani et al., 2007). Postprandial LPS, packaged within chylomicrons, engages Toll-like receptor 4 (TLR4) on Kupffer cells, hepatocytes, and metabolic tissues to drive low-grade inflammation, hepatic steatosis, atherogenic dyslipidemia, and impaired insulin signaling (Cani et al., 2007; Clemente-Postigo et al., 2012; Mohammad and Thiemermann, 2020). LPS–TLR4 activation converges on lipid-sensitive transcriptional networks, including SREBP and liver X receptor (LXR), and interfaces with peroxisome proliferator-activated receptor-α (PPARα), thereby coupling innate immune sensing of gut microbiota–derived signals to hepatic lipid and glucose metabolism (Castrillo et al., 2003). Although both feeding-driven LPS–TLR4 activation and statins act directly on SREBP-regulated cholesterol biosynthesis, and feeding–fasting cycles are major regulators of both SREBP activity and metabolic endotoxemia, it remains unknown how feeding-induced LPS–TLR4 signaling interacts with statin actions on the SREBP-regulated cholesterol biosynthetic pathway, and whether such crosstalk contributes to the context-dependent metabolic effects of statins.

To address these knowledge gaps, we designed two complementary simvastatin dosing regimens in wild-type mice: (1) delivery by oral gavage during the fasting (sleep) phase and (2) incorporation into chow during the active feeding phase. In this context, feeding-phase simvastatin lowered HDL-C and improved glucose homeostasis, whereas fasting-phase simvastatin increased HDL-C but impaired glucose tolerance, revealing a striking nutritional-state dependence of simvastatin’s metabolic actions. We then leveraged Tlr4-deficient mice, a canonical LPS-signaling–defective model, to interrogate the contribution of metabolic endotoxemia (Poltorak et al., 1998). These experiments identify a feeding-induced surge in TLR4-dependent LPS signaling as a key modulator of the LXR/SREBP-1c/PPARα axis, thereby mechanistically linking nutritional state and metabolic endotoxemia to simvastatin’s divergent effects on HDL metabolism and glucose regulation.

## 2 Materials and methods

### 2.1 Animals

Male A/J, C3H/HeJ, and C3H/HeOuJ mice were obtained from The Jackson Laboratory (Bar Harbor, ME, United States) and housed at 22 °C ± 2 °C on a 12-h light/dark cycle (lights on at 6:00 a.m. [ZT0], lights off at 6:00 p.m. [ZT12]). Unless otherwise specified, mice had *ad libitum* access to standard chow and water. All procedures conformed to the NIH *Guide for the Care and Use of Laboratory Animals* and were approved by the University of Maryland, Baltimore Institutional Animal Care and Use Committee (IACUC protocol #AUP-00001103). The study was designed and reported in accordance with ARRIVE guidelines.

### 2.2 Simvastatin administration

To investigate the differential effects of simvastatin in fasting versus feeding states, mice were maintained on standard low-fat chow, under which they typically consume ∼80% of their daily food during the dark (active) phase (ZT12–ZT24) and very little during the light (rest) phase (ZT0–ZT12) (Mukherji et al., 2019; Turek et al., 2005). Two complementary dosing regimens were used: oral gavage during the light-phase fasting period (“fasting-phase” dosing) and dietary admixture during the dark-phase feeding period (“feeding-phase” dosing).

#### 2.2.1 Fasting-phase dosing (oral gavage)

Ten male A/J mice (8–10 weeks old, ∼20–25 g) were randomly assigned to receive either simvastatin (n = 5) or vehicle (n = 5). Simvastatin (MedChemExpress, #HY-17502; purity: 99.56%) was suspended in 1% methylcellulose (MedChemExpress, #HY-125861A) prepared in 1× PBS and administered once daily by oral gavage at 10:00 a.m. (ZT04) at 16 mg/kg body weight using 20-gauge plastic feeding tubes. This time point falls within the light-phase fasting period, modeling statin administration during relative caloric deprivation. The 16 mg/kg dose was chosen as an intermediate, well-tolerated dose within the 10–100 mg/kg range widely used in mouse studies and has been shown to elicit robust pharmacodynamic effects without overt toxicity in models of neuroinflammation, lipid metabolism, and muscle physiology (Esposito et al., 2012; He et al., 2017; Sparrow et al., 2001; Zhang et al., 2024). This dose is also within the range that approximates high-intensity clinical dosing after body-surface-area scaling. Mice were treated for 5 weeks. Plasma and tissue samples were collected at ZT09 (3:00 p.m.), 5 hours after gavage, to capture the metabolic state associated with fasting-phase simvastatin exposure.

#### 2.2.2 Feeding-phase dosing (dietary administration)

For feeding-phase studies, ten male mice (8–10 weeks old, ∼20–25 g) of each strain—A/J, TLR4-deficient C3H/HeJ, and TLR4 wild-type control C3H/HeOuJ—were randomly assigned to either control diet (n = 5 per strain) or simvastatin-supplemented diet (n = 5 per strain). Control mice received standard chow (Diet D1001; 10 kcal% fat, 20 kcal% protein, 70 kcal% carbohydrate; Research Diets, New Brunswick, NJ), whereas treated mice received chow containing simvastatin (0.1 g/kg food weight; Formulation D11060903i, Research Diets) for 5 weeks (Zhang et al., 2024). Assuming an average 25 g mouse consumes ∼4 g of chow per day, this formulation delivers ∼0.4 mg simvastatin per 25 g body weight (∼16 mg/kg/day). Using body-surface-area conversion (mouse-to-human factor 12.3), this corresponds to a human-equivalent dose of ∼1.3 mg/kg, approximating an 80 mg/day regimen in a 60-kg adult (Reagan-Shaw et al., 2008; Zhang et al., 2024). This high-intensity dose has been associated with increased risk of myopathy and new-onset diabetes in humans and was therefore selected to interrogate mechanisms underlying both efficacy and adverse metabolic effects of simvastatin (Zhang et al., 2024). For dietary studies, plasma and tissues were collected at ZT05 (11:00 a.m.), approximately 5 hours after peak nocturnal food intake, to reflect the postprandial feeding state.

Throughout simvastatin treatment, animals were monitored daily for health and welfare; no unexpected deaths or exclusions occurred before the planned experimental endpoints. Investigators performing biochemical assays and data analyses were blinded to group allocation.

### 2.3 Glucose tolerance test (GTT)

Six days before completion of the 5-week simvastatin or control treatment, glucose tolerance was assessed. For the fasting-phase cohort, A/J mice were fasted for 5 h after their daily oral gavage and then subjected to GTT at ZT09 (3:00 p.m.). For the feeding-phase cohort, A/J, C3H/HeJ, and C3H/HeOuJ mice were fasted for 5 h after removal of food, and GTT was performed at ZT05 (11:00 a.m.). Baseline fasting blood glucose was measured from tail-vein blood prior to glucose administration. Mice then received an intraperitoneal injection of glucose (2 g/kg body weight), and blood glucose concentrations were measured from tail-vein samples at 15, 60, 120, and 180 min using an AlphaTRAK glucometer (Zoetis, Parsippany, NJ). Glucose tolerance was quantified as the area under the curve (AUC) using trapezoidal integration (Solberg et al., 2006; Zhang et al., 2024).

### 2.4 Glycerol tolerance test

Three days before completion of the 5-week simvastatin or control treatment, glycerol tolerance was evaluated in the same cohorts. For the fasting-phase cohort, A/J mice were fasted for 5 h after their daily oral gavage and tested at ZT09 (3:00 p.m.). For the feeding-phase cohort, A/J, C3H/HeJ, and C3H/HeOuJ mice were fasted for 5 h after removal of food, and testing was performed at ZT05 (11:00 a.m.). Baseline fasting blood glucose was measured from tail-vein blood, after which mice received an intraperitoneal injection of glycerol (1.5 g/kg body weight). Plasma glucose levels were measured from tail-vein blood at designated time points after injection to assess glycerol tolerance (Kim et al., 2017).

### 2.5 Biochemical measurements

Plasma cholesterol fractions (HDL-C, total cholesterol [TC], LDL-C, unesterified cholesterol [UC]) and triglycerides were quantified using enzymatic kits (MilliporeSigma) according to the manufacturer’s protocols. Reference calibrators supplied with these kits were analytical grade with certified purity ≥95% according to the manufacturers’ certificates of analysis.

### 2.6 Hepatic lipid analysis

Liver lipids were extracted using a modification of the Bligh and Dyer method (Zhang et al., 2019). Approximately 100 mg of liver tissue was homogenized in 4 mL of chloroform/methanol (2:1) and incubated overnight at room temperature. After adding 800 µL of 0.9% saline, samples were centrifuged at 2,000 g for 10 min. The organic phase was collected, dried under vacuum, and dissolved in butanol/(Triton X-100/methanol [2:1]) (30:20). Triglycerides were quantified using a colorimetric assay (Sigma), and free fatty acids were measured using FFA kits (Wako Chemicals). All lipid standards used for calibration were analytical grade with manufacturer-certified purity ≥95%.

### 2.7 24(S)-hydroxycholesterol quantification

Hepatic 24(S)-hydroxycholesterol (24S-HC) levels were measured using a 24(S)-hydroxycholesterol ELISA kit (Abcam, #ab204530). Approximately 200 mg of liver tissue was homogenized in 3 mL of 95% ethanol. The homogenate was centrifuged, pellets were resuspended in 1 mL ethanol/dichloromethane (1:1, v/v), and the mixture was centrifuged again. Supernatants from both steps were combined and evaporated to dryness using a rotary evaporator. Dried extracts were reconstituted in 16 μL 95% ethanol and 484 μL assay buffer, then diluted as needed in assay buffer before ELISA. The 24S-HC reference standard supplied with the kit has a purity ≥95% per the manufacturer’s specifications and was used to generate the calibration curve.

### 2.8 Cell culture

Huh-7 human hepatoma cells, originally provided by Dr. Liqing Yu, were maintained at 37 °C in a humidified incubator with 5% CO_2_. Cells were grown in Dulbecco’s Modified Eagle Medium (DMEM) supplemented with 10% fetal bovine serum (FBS), 100 U/mL penicillin, and 100 μg/mL streptomycin and were used for experiments at subconfluence.

For fasting-mimetic conditions (Figures 3A,B,E; Supplementary Figure S3), cells were washed twice with 1× PBS, incubated overnight in serum-free DMEM, and then treated for 24 h with 5 µM simvastatin alone or in combination with one or more of the following: 50 µM oleic acid (MilliporeSigma; C18:1, #O1383, purity >99% by GC); 10 µM GW6471 (MedChemExpress; #HY-15372, purity 99%), a selective PPARα antagonist; 500 µM leucine (MedChemExpress; #HY-N0486, purity 98%), which suppresses autophagy initiation via mTORC1 activation; or 50 µM leupeptin (MedChemExpress; #HY-18234A, purity 99.39%), a cysteine/serine/threonine protease inhibitor that blocks autophagic flux (Chen et al., 2021; Desideri et al., 2023; Sun et al., 2021).

For serum-replete conditions (Figures 4A,C), cells were maintained in DMEM containing 10% FBS and treated for 8 h with 50 µM oleic acid, 250 µM leucine, or 10 nM insulin, with or without 10 µM simvastatin. After treatment, total RNA was isolated using TRIzol reagent according to the manufacturer’s instructions.

### 2.9 RNA extraction and quantitative real-time PCR

Liver tissues were snap-frozen in liquid nitrogen at the time of dissection, stored at −80 °C, then cut into ∼50-mg pieces on dry ice and homogenized in TRIzol reagent (Invitrogen) using a motorized tissue homogenizer. Total RNA was extracted and purified according to the manufacturer’s instructions, and 1–3 µg of RNA were used for cDNA synthesis with the iScript™ Advanced cDNA Synthesis Kit (Bio-Rad). Quantitative real-time PCR was performed with iTaq™ Universal SYBR® Green Supermix (Bio-Rad) on a StepOnePlus (Applied Biosystems) or CFX96 (Bio-Rad) instrument.

Target transcripts included genes involved in cholesterol and HDL metabolism, autophagy, and SREBP/LXR/PPARα signaling (e.g., *Apoa1, Apoa2, Pltp, Srebf2, Sqle, Lxrα, Ppara*, and others as listed in Supplementary Table S1). For mouse tissues, expression levels were normalized to the housekeeping genes *B2m* and *Hprt*; for human Huh-7 cells, normalization was performed to *B2M* and *HPRT1*. Relative mRNA abundance was calculated using the ΔΔCt method.

### 2.10 Statistical analysis

Statistical analyses were performed using Prism (GraphPad Software, San Diego, CA). Two-way ANOVA was applied for experiments involving multiple groups, followed, when appropriate, by pairwise Student’s t-tests when significant interactions were detected (p < 0.05). Exact n values for each group are provided in the figure legends. Data are presented as mean ± standard error of the mean (SEM), and significance levels from pairwise comparisons are indicated in the figures.

## 3 Results

### 3.1 Fasting versus feeding: divergent effects of simvastatin on HDL and glucose metabolism

**FIGURE 1:**
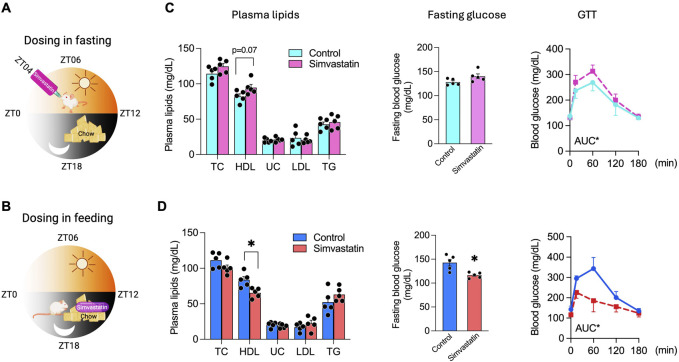
Differential Effects of Simvastatin Administration during Fasting or Feeding on HDL Cholesterol and Glucose Metabolism. **(A,B)** Schematic representations of the 5-week simvastatin treatment regimens. Mice were housed on a 12-h light/dark cycle (lights on at 06:00, ZT0; lights off at 18:00, ZT12). In **(A)**, simvastatin was delivered by oral gavage at ZT04 (10:00 a.m.) during the light-phase fasting period. In **(B)**, simvastatin was incorporated into standard chow, allowing consumption during the dark-phase feeding period. **(C)** Plasma lipid and glucose parameters in mice receiving fasting-phase simvastatin (gavage), including high-density lipoprotein cholesterol (HDL-C), total cholesterol (TC), unesterified cholesterol (UC), triglycerides (TG), fasting glucose, and area under the curve (AUC) for glucose tolerance tests (GTTs). **(D)** Plasma lipid and glucose parameters in mice receiving feeding-phase simvastatin (chow admixture), measured as in **(C)**. Data are presented as mean ± SEM (n = 5 per group). Statistical significance was determined by one-way ANOVA: *p* < 0.05.

Under standard chow conditions, mice consume approximately 80% of their food during the dark (active) feeding phase (ZT12–ZT24) and minimally during the light (rest) fasting phase (ZT0–ZT12) (Mukherji et al., 2019; Turek et al., 2005). Capitalizing on this natural diurnal rhythm, we investigated whether fasting versus feeding states would differentially modulate simvastatin’s lipid and glucose regulatory effects in A/J mice over 5 weeks. We used two dosing strategies: simvastatin incorporated into chow (0.1 g/kg food weight; formulation D11060903i, Research Diets), ensuring drug ingestion during the active feeding phase, and oral gavage of an equivalent dose (16 mg/kg body weight) at 10:00 a.m. (ZT04), coinciding with the light-phase fasting period (Figures 1A,B). A typical 25-g mouse consumes approximately 3–5 g of food daily, corresponding to about 16 mg/kg body weight, which aligns with the orally gavaged dose. This approach preserved normal feeding rhythms and allowed a direct comparison of simvastatin’s effects when co-administered with food versus during fasting. Mice were housed on a 12-h light/dark cycle (lights on at 6:00 a.m. [ZT0] and off at 6:00 p.m. [ZT12]) with *ad libitum* access to food and water. The 16 mg/kg dose was chosen to approximate an 80 mg human equivalent daily dose (Zhang et al., 2024), maintaining translational relevance.

After 5 weeks, we collected plasma and tissues at two time points to capture the drug’s effects in distinct metabolic states: the fasting-state group (ZT09, 3:00 p.m.), 5 hours after the last oral gavage, and the feeding-state group (ZT05, 11:00 a.m.), 5 hours after the active eating period. Mice receiving simvastatin by gavage during fasting showed a modest increase in HDL-C (p = 0.07), with no significant changes in total cholesterol (TC), unesterified cholesterol (UC), or triglycerides (TG) (Figure 1C). Low-density lipoprotein cholesterol (LDL-C) also remained unchanged, consistent with the characteristically low LDL-C in rodents (which lack cholesteryl ester transfer protein, CETP) (Steenbergen et al., 2010). Notably, fasting-phase simvastatin markedly impaired glucose metabolism, as evidenced by higher glucose area under the curve (AUC) values in glucose tolerance tests (GTT) in 6 days before completion of the 5-week simvastatin or control treatment (Figure 1C). In contrast, co-administration of simvastatin with food significantly lowered HDL-C levels and improved glucose homeostasis, indicated by reduced fasting blood glucose and enhanced glucose tolerance (Figure 1D).

To determine whether these time-of-day–dependent effects are restricted to a single sampling point or reflect broader alterations in circadian lipid homeostasis, we next profiled 24-h plasma lipid dynamics in a separate cohort. Serial sampling across the light period (0:00, 6:00, 12:00, and 18:00 h) revealed robust nyctohemeral variation in HDL-C, and confirmed that simvastatin given in the fasting state versus with food produced consistently opposite effects on HDL-C throughout the daytime interval (06:00–18:00) (Supplementary Figure S1). Fasting-phase simvastatin shifted HDL-C upward relative to controls, whereas feeding-phase simvastatin shifted HDL-C downward across these time points, reinforcing the concept that dosing in the fasted versus fed state drives divergent HDL responses over the entire active phase rather than at a single clock time. Together, these data indicate that administering simvastatin during fasting versus feeding exerts opposing effects on HDL and glucose metabolism.

We also assessed whether these interventions altered the hepatic molecular clock. Hepatic mRNA levels of two core clock genes, *Bmal1*(Arntl) and *Per2*, were measured in all four primary groups (fasting-vehicle, fasting-simvastatin, feeding-vehicle, feeding-simvastatin) (Supplementary Figure S2). *Bmal1* and *Per2* displayed the expected anti-phasic pattern in relation to the light–dark cycle (Greco et al., 2021), but their expression did not differ substantially between vehicle and simvastatin within a given nutritional state. These findings suggest that our dosing protocols did not markedly re-phase the hepatic clock and support the conclusion that feeding status—rather than disruption of intrinsic circadian machinery—is the dominant driver of the divergent metabolic responses to simvastatin.

### 3.2 Fasting and feeding states govern Simvastatin’s regulation of PPARα

**FIGURE 2:**
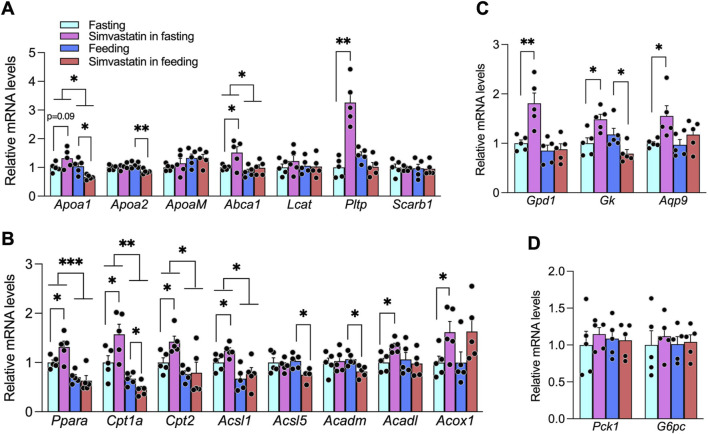
Hepatic gene expression modulation by simvastatin in fasting and feeding phases. **(A)** Relative hepatic mRNA levels of HDL-related genes (Apoa1, Apoa2, Apom, Abca1, Lcat, Pltp, and Scarb1). Liver samples were collected 5 h after the last dose—ZT09 (15:00) for the fasting/gavage regimen and ZT05 (11:00) for the feeding/chow regimen. **(B)** Hepatic expression of Pparα and its downstream fatty acid oxidation targets (Cpt1a, Cpt2, Acsl1, Acadl, Acox1, Acadm, Acsl5). **(C)** Expression of Pparα-dependent glycerol–gluconeogenesis genes (Gpd1, Gk, Aqp9). **(D)** Expression of gluconeogenic genes in the pyruvate/lactate pathway (Pck1 and G6pc). Bars represent mean ± SEM (n = 5 per group). All qRT-PCR data were normalized to Hprt. Statistical significance is indicated by *p* < 0.05; *p < 0.01; **p < 0.001 (two-way ANOVA with Tukey’s *post hoc* test).

To determine whether the opposing effects of simvastatin on circulating HDL-C reflect coordinated changes in HDL biogenesis, we prospectively quantified hepatic expression of genes involved in HDL synthesis (apolipoprotein A1 [*Apoa1*], *Apoa2*, *Apom*, and ATP-binding cassette subfamily A member 1 [*Abca1*]), HDL maturation (lecithin–cholesterol acyltransferase [*Lcat*] and phospholipid transfer protein [*Pltp*]), and HDL clearance (scavenger receptor class B type 1 [*Scarb1*], encoding SR-B1) (Paththinige et al., 2017). When simvastatin was administered during fasting, *Pltp* expression increased significantly and *Apoa1* showed a modest increase (p < 0.1), whereas *Apoa2*, *Apom*, *Abca1*, *Lcat*, and *Scarb1* remained unchanged (Figure 2A). By contrast, simvastatin administration during feeding suppressed both *Apoa1* and *Apoa2*. Thus, our *a priori* analysis of HDL pathway genes confirms that fasting- and feeding-phase simvastatin differentially remodel the hepatic transcriptional program governing HDL synthesis and remodeling, consistent with the divergent HDL-C phenotypes (Figures 1C,D, 2A).

Because *Apoa1*, *Apoa2*, and *Pltp* are regulated by PPARα ligands such as fibrates and because statins can activate PPARα to induce *APOA1* in human cells, we hypothesized that nutritional state would dictate how simvastatin engages PPARα to control these HDL-related genes (Bouly et al., 2001; Han et al., 2006; Martin et al., 2001). Consistent with this model, fasting-phase simvastatin significantly upregulated hepatic PPARα and its canonical targets, including carnitine palmitoyltransferase 1A (*Cpt1a*), carnitine palmitoyltransferase 2 (*Cpt2*), acyl-CoA synthetase long-chain family member 1 (*Acsl1*), acyl-CoA dehydrogenase long chain (*Acadl*), and acyl-CoA oxidase 1 (*Acox1*). In contrast, simvastatin co-administered with food reduced the expression of several key PPARα targets, including *Cpt1a*, acyl-CoA dehydrogenase medium chain (*Acadm*), and acyl-CoA synthetase long-chain family member 5 (*Acsl5*) (Figure 2B). Together with the *Apoa1*, *Apoa2*, and *Pltp* data (Figure 2A), these findings indicate that fasting and feeding states critically shape simvastatin-mediated PPARα activation and, in turn, transcriptional regulation of HDL synthesis genes.

Beyond lipid handling, PPARα plays a major role in glucose homeostasis, particularly by promoting glycerol-fueled gluconeogenesis (Patsouris et al., 2004). To evaluate how nutritional state modifies this axis, we examined PPARα-responsive genes in the glycerol pathway. Fasting-phase simvastatin significantly enhanced hepatic expression of glycerol-3-phosphate dehydrogenase 1 (*Gpd1*), glycerol kinase (*Gk*), and aquaporin 9 (*Aqp9*), whereas feeding-phase simvastatin decreased their expression (Figure 2C). In contrast, expression of core gluconeogenic enzymes phosphoenolpyruvate carboxykinase 1 (*Pck1*) and glucose-6-phosphatase catalytic subunit (*G6pc*) was unchanged by simvastatin in either nutritional state (Figure 2D). Thus, simvastatin dosing during fasting versus feeding differentially modulates PPARα-dependent glycerol utilization without directly altering the gluconeogenic program, providing a mechanistic basis for the distinct glycemic responses observed *in vivo* (Figures 1C,D).

To further test whether PPARα activation is required for simvastatin’s effects on HDL-related genes and glycerol metabolism, we treated Huh-7 hepatoma cells with simvastatin in the presence or absence of the selective PPARα antagonist GW6471 (Sun et al., 2021). Simvastatin alone robustly increased *APOA1* and *PLTP* mRNA, as well as the glycerol pathway gene *GK*, whereas co-treatment with GW6471 largely abolished these responses; GW6471 alone had minimal effects (Supplementary Figure S3). These pharmacologic data support a causal role for PPARα in mediating simvastatin-induced upregulation of HDL synthesis genes and glycerol-handling enzymes in hepatocytes, reinforcing the *in vivo* conclusion that PPARα links nutritional state to simvastatin’s effects on HDL-C and glucose homeostasis.

### 3.3 SREBP-2-driven autophagy links simvastatin to PPARα activation

**FIGURE 3:**
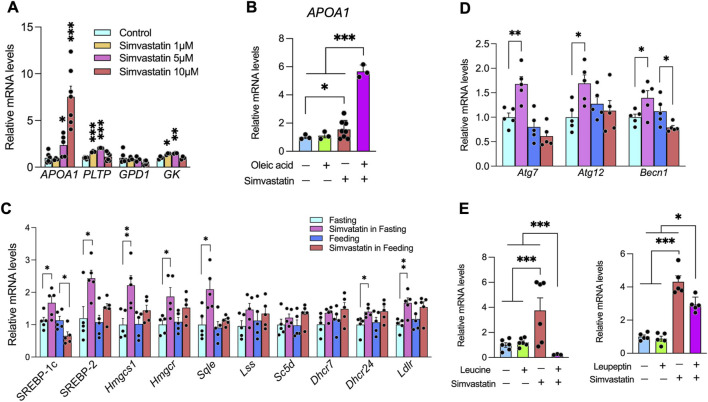
Simvastatin administration during fasting activates SREBP-2 and autophagy and augments FFA-induced *APOA1* expression. **(A)** Huh7 cells cultured under serum-depleted conditions were treated with simvastatin (1, 5, or 10 μM) for 24 h. APOA1, PLTP, and GK mRNA levels were quantified by qPCR, normalized to the geometric mean of *HPRT* and *B2M*, and expressed as fold change relative to untreated controls. **(B)** Simvastatin (10 μM) enhances sodium oleate (50 μM)–induced *APOA1* expression in serum-depleted Huh7 cells. Gene expression was measured by qPCR and normalized to *HPRT* and *B2M*. **(C)** Fasting-phase simvastatin treatment upregulates hepatic *Srebf2* and its target genes *in vivo*. A/J mice received simvastatin by gavage for 5 weeks and were sacrificed 5 h after the final dose (ZT09); hepatic mRNA levels were normalized to *Hprt*. **(D)** Expression of autophagy-related genes (*Atg7*, *Atg12*, *Becn1* and others) in liver following fasting-phase simvastatin treatment. **(E)** Pharmacologic inhibition of autophagy attenuates simvastatin-induced *APOA1* expression in Huh7 cells. Serum-starved cells were treated with simvastatin in the presence or absence of 500 µM leucine (mTORC1 activator) or 50 µM leupeptin (lysosomal protease inhibitor); *APOA1* mRNA was measured by qPCR and normalized to *HPRT1* and *B2M*. Data are mean ± SEM with individual data points overlaid (n indicated in each panel). Statistical significance: *p* < 0.05; *p < 0.01; **p < 0.001 (two-way ANOVA with Tukey’s *post hoc* test).

We next asked how simvastatin activates PPARα under fasting-like conditions. Serum-starving Huh-7 cells overnight and then exposing them to increasing doses of simvastatin led to a dose-dependent induction of *PLTP*, *APOA1*, and *GPD1* (Figure 3A), mirroring the fasting-phase effects observed *in vivo* (Figure 2A). Although free fatty acids (FFAs) generated by fasting-induced adipose lipolysis are known PPARα ligands (Bideyan et al., 2021), treatment of Huh-7 cells with oleic acid alone did not increase *PLTP*, *APOA1*, or *GPD1* expression, suggesting that FFAs require additional signals to fully engage PPARα. Notably, when oleic acid was combined with simvastatin, *APOA1* expression was markedly higher than with simvastatin alone (Figure 3B), indicating that simvastatin can potentiate FFA-mediated activation of the PPARα–HDL gene program.

Autophagy has emerged as a key facilitator of PPARα signaling by degrading nuclear corepressors such as NCoR1 and HDAC3 (Iershov et al., 2019; Saito et al., 2019), and SREBP-2 transactivation can itself trigger autophagy (Seo et al., 2011). In liver from fasting mice, simvastatin markedly increased SREBP-2 expression and its downstream cholesterol synthesis targets by ∼1–3-fold (Figure 3C), coincident with upregulation of key autophagy-related genes, including *Atg7*, *Atg12*, and *Becn1* (Figure 3D). These findings suggest that simvastatin activates a SREBP-2–dependent autophagy program under fasting conditions.

To directly test whether autophagic flux is required for simvastatin’s transcriptional effects, serum-starved Huh-7 cells were treated with simvastatin together with pharmacologic inhibitors of autophagy. Leucine, which activates mTORC1 and suppresses autophagy (Chen et al., 2021), completely abolished simvastatin-induced *APOA1* expression (Figure 3E, left). Leupeptin, a cysteine/serine/threonine protease inhibitor that blocks lysosomal degradation and causes accumulation of autolysosomes (Desideri et al., 2023; Yang et al., 2013), significantly attenuated—but did not fully abolish—the simvastatin-mediated increase in *APOA1* mRNA (Figure 3E, right). Thus, inhibition of either the initiation phase of autophagy (via mTORC1 activation) or the degradative phase (via lysosomal blockade) markedly blunts simvastatin-induced *APOA1* upregulation, supporting a requirement for intact autophagic flux. Collectively, these data support a model in which simvastatin triggers SREBP-2–dependent autophagy during fasting, thereby enhancing FFA-mediated PPARα activation and ultimately modulating HDL levels and hepatic glucose metabolism *in vivo*.

### 3.4 Postprandial LPS undermines Simvastatin’s activation of PPARα

**FIGURE 4:**
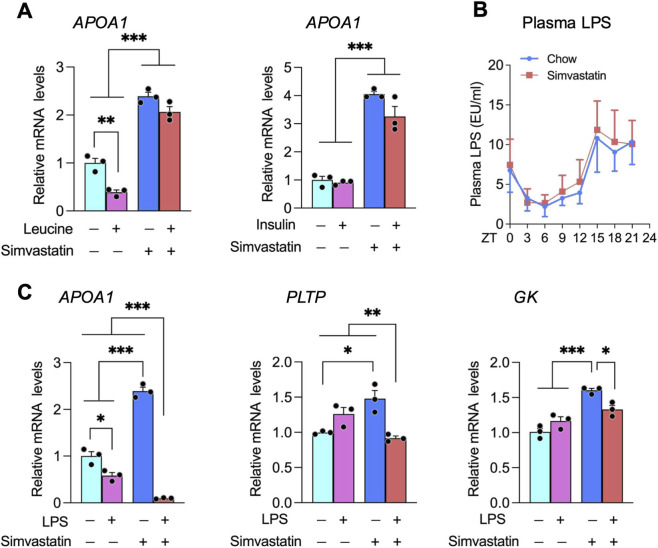
Interplay between lipopolysaccharide (LPS) and simvastatin in regulating *APOA1*, *PLTP*, and *GK* expression. **(A)** Effect of leucine and insulin co-treatment on simvastatin-induced *APOA1* expression in Huh7 cells. Cells were treated under the indicated conditions, and *APOA1* mRNA levels were quantified by qPCR. **(B)** Plasma LPS concentrations in mice across the light (ZT0–12; fasting) and dark (ZT12–24; feeding) phases, illustrating alignment of transient endotoxemia with feeding behavior. **(C)** Effect of LPS co-treatment on simvastatin-regulated *APOA1*, *PLTP*, and *GK* expression in Huh7 cells. Gene expression was quantified by qPCR and normalized to the geometric mean of *HPRT1* and *B2M*. Data are presented as mean ± SEM with individual points shown (n indicated in each panel). Statistical significance: *p* < 0.05; *p < 0.01; **p < 0.001 (two-way ANOVA with Tukey’s *post hoc* test).

Co-administration of simvastatin with food suppressed PPARα activation, contrasting with the enhanced activation observed when simvastatin was given during fasting (Figures 2B,C). To identify feeding-associated factors that could antagonize PPARα, we first tested common nutrients and hormones in Huh-7 cells under serum-replete conditions (10% FBS). Neither leucine nor insulin altered simvastatin-induced *APOA1* expression (Figure 4A), and neither oleic acid nor high glucose affected simvastatin’s transcriptional effects (data not shown). These observations suggest that typical postprandial nutrient and insulin signals are insufficient to explain the attenuation of PPARα activation by feeding.

Recent work instead implicates postprandial absorption of gut microbiota–derived LPS in transient elevations of circulating LPS, a phenomenon termed metabolic endotoxemia (Cani et al., 2007; Mohammad and Thiemermann, 2020). LPS can be incorporated into intestinally secreted lipoproteins such as chylomicrons and thereby enter the systemic circulation (Clemente-Postigo et al., 2012). We therefore measured plasma LPS levels during fasting (ZT03–09) and feeding (ZT15–21) and found that, irrespective of simvastatin treatment, circulating LPS peaked during feeding and was significantly lower during fasting (Figure 4B). To test whether LPS directly modulates simvastatin’s actions, we co-treated Huh-7 cells with simvastatin and LPS under serum-replete conditions. LPS co-treatment abolished simvastatin-induced upregulation of *PLTP*, *APOA1*, and *GK* (Figure 4C). These findings indicate that microbiota-derived LPS, which rises with feeding, can antagonize simvastatin-driven PPARα activation and HDL/glycerol gene expression, providing a mechanistic explanation for the diminished PPARα signaling observed when simvastatin is administered in the fed state (Figures 2A–C).

### 3.5 LPS-statin interaction: a key determinant of oxysterol metabolism in the liver

**FIGURE 5:**
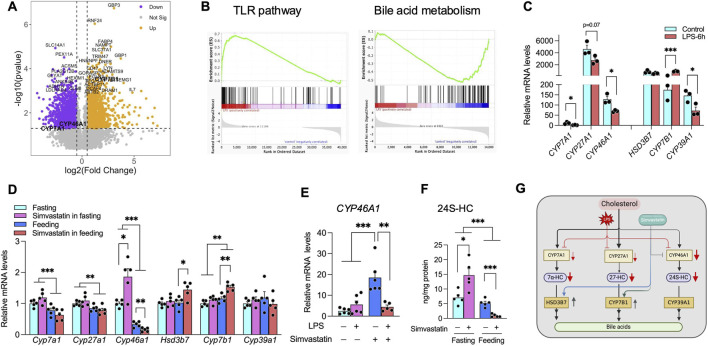
LPS–statin interaction is a key determinant of hepatic oxysterol metabolism. **(A)** Volcano plot of differentially expressed genes (protein-coding and non-coding) in LPS-treated differentiated HepaRG (dHepaRG) cells (GEO: GSE230325). A total of 43,892 genes were detected. Key oxysterol-related enzymes (*CYP7A1*, *CYP46A1*, *CYP7B1*) are highlighted. The x-axis shows log2 fold change; the y-axis shows −log10 *p*-value. Dashed lines denote thresholds for significance (*p* < 0.05; |log2 fold change| > 1). **(B)** Gene Set Enrichment Analysis (GSEA) of LPS-regulated genes. Pathways enriched among LPS-upregulated genes include infection and Toll-like receptor (TLR) signaling, whereas LPS-downregulated genes are enriched for bile acid metabolism, xenobiotic processing, and fatty acid metabolism. **(C)** qPCR validation of oxysterol-metabolism genes in LPS-treated dHepaRG cells. LPS lowers expression of oxysterol-producing enzymes (*CYP7A1*, *CYP27A1*, *CYP46A1*) and increases *CYP7B1*, a 27-hydroxycholesterol–metabolizing enzyme. **(D)** Feeding-phase simvastatin further remodels bile acid/oxysterol pathways in mouse liver. qPCR analyses show that feeding reduces *Cyp7a1*, *Cyp27a1*, and *Cyp46a1*, while simvastatin co-administration augments expression of oxysterol-catabolizing enzymes (*Cyp7b1*, *Hsd3b7*). **(E)** In Huh7 cells, simvastatin robustly upregulates *CYP46A1* mRNA, an effect markedly attenuated by LPS co-treatment. **(F)** Hepatic 24(S)-hydroxycholesterol (24S-HC) levels in mice treated with simvastatin under fasting or feeding conditions. Simvastatin increases 24S-HC in the fasting regimen but decreases 24S-HC when administered with food. **(G)** Working model depicting how feeding-driven LPS signaling converges with simvastatin to deplete hepatic oxysterols by suppressing the classic (*CYP7A1*-mediated) and acidic (*CYP27A1*, *CYP46A1*) pathways and upregulating oxysterol-metabolizing *CYP7B1*, leading to reduced LXR activity. Data are mean ± SEM with individual data points shown (n indicated in each panel). Statistical significance was assessed by one-way ANOVA for **(C)** and by two-way ANOVA with Tukey’s *post hoc* test for the remaining panels: *p* < 0.05; *p < 0.01; **p < 0.001.

To investigate how feeding-driven LPS might modulate simvastatin’s regulation of PPARα activity—which governs fasting glucose and HDL metabolism—we reanalyzed transcriptomic data from differentiated HepaRG (dHepaRG) cells treated with LPS for 6 hours (GEO: GSE230325) (Ehle et al., 2024). dHepaRG cells retain key hepatic features, including hepatocyte-like morphology and the expression of metabolic enzymes, nuclear receptors, and drug transporters (Ehle et al., 2024). Our analysis covered both protein-coding and non-coding RNAs, with 43,892 genes detected in total; a volcano plot revealed significant transcriptional changes (Figure 5A).

Gene Set Enrichment Analysis (GSEA) of LPS-upregulated genes showed enrichment in pathways related to infection, cytokine-receptor interactions, and Toll-like receptor signaling—specifically involving the LPS receptor TLR4 (Figure 5B). By contrast, LPS-downregulated genes were enriched in pathways tied to bile acid metabolism, xenobiotic and fatty acid metabolism, peroxisomal functions, and estrogen response (Chen et al., 2013; Kuleshov et al., 2016; Mootha et al., 2003; Subramanian et al., 2005; Xie et al., 2021). We focused on two salient changes: the reduction in bile acid metabolism-related genes and the induction of TLR signaling, aligning with previous reports that LPS suppresses bile acid synthesis and that statins also influence bile acid metabolism (Khovidhunkit et al., 2004; Schonewille et al., 2016). Additionally, LPS-activated TLR4 impacts lipid metabolism, including bile acid pathways (Castrillo et al., 2003).

In the liver, cholesterol is converted to bile acids through two major routes. In the classic (neutral) pathway, cholesterol 7α-hydroxylase (*CYP7A1*) catalyzes the rate-limiting step to generate 7α-hydroxycholesterol (7α-HC), which is further processed by 3β-hydroxy-Δ^5^-C_27_-steroid oxidoreductase (*HSD3B7*) to form cholic acid and chenodeoxycholic acid (CDCA) (Pandak and Kakiyama, 2019). In the acidic pathway, sterol 27-hydroxylase (*CYP27A1*) produces (25R)26-hydroxycholesterol (27-HC), which—together with other oxysterols—is further hydroxylated by oxysterol 7α-hydroxylase (*CYP7B1*) en route to CDCA (Pandak and Kakiyama, 2019). Cholesterol 24(S)-hydroxylase (*CYP46A1*) generates 24(S)-hydroxycholesterol (24S-HC), best characterized in neurons but also expressed in liver, where it exerts regulatory effects on lipid metabolism (Si et al., 2020). In LPS-treated dHepaRG cells, expression of *CYP7A1*, *CYP46A1*, and *CYP39A1* was markedly reduced, with a more modest decline in *CYP27A1* (p = 0.07), whereas *CYP7B1* was induced more than fivefold (Figure 5C). These data suggest that LPS not only suppresses the classic pathway but also reshapes the acidic pathway by downregulating oxysterol-producing enzymes (*CYP27A1*, *CYP46A1*) while upregulating the oxysterol-metabolizing enzyme *CYP7B1*, thereby favoring depletion of regulatory oxysterols such as 27-HC.

We next examined how bile acid and oxysterol metabolism are altered *in vivo* when simvastatin is administered during the feeding phase, a context in which circulating LPS is elevated (Figure 4B). Relative to fasting, feeding alone decreased hepatic expression of the oxysterol-producing enzymes *Cyp7a1*, *Cyp27a1*, and *Cyp46a1* (Figure 5D). Feeding-phase simvastatin further increased the expression of the oxysterol-catabolizing enzymes *Hsd3b7* and *Cyp7b1* (Figure 5D). Although *Cyp39a1* was unchanged, feeding-phase simvastatin further suppressed *Cyp46a1*, in contrast to the upregulation observed under fasting-phase simvastatin treatment (Figure 5D), indicating that feeding-related signals can override simvastatin-induced *Cyp46a1* expression.

Consistent with these *in vivo* findings, simvastatin markedly increased *CYP46A1* expression in Huh-7 cells, an effect that was strongly attenuated by co-treatment with LPS (Figure 5E). In mouse liver, direct quantification of 24(S)-hydroxycholesterol (24S-HC) using an ELISA kit showed that simvastatin decreased 24S-HC levels when administered with food, whereas it increased 24S-HC under fasting conditions (Figure 5F). Taken together, these findings indicate that feeding-driven LPS signaling remodels hepatic oxysterol metabolism—suppressing oxysterol synthesis while enhancing oxysterol catabolism—such that simvastatin given in the fed state depletes hepatic oxysterol pools (Figure 5G). Because these oxysterols serve as endogenous ligands for LXR and thereby regulate *Apoa1*, *Apoa2*, and *Pltp* expression, this LPS–statin interaction provides an upstream mechanism by which nutritional state and metabolic endotoxemia shape simvastatin’s impact on HDL synthesis and PPARα-dependent glucose regulation.

### 3.6 Feeding-phase simvastatin depletes oxysterols and suppresses LXR–SREBP-1c signaling

**FIGURE 6:**
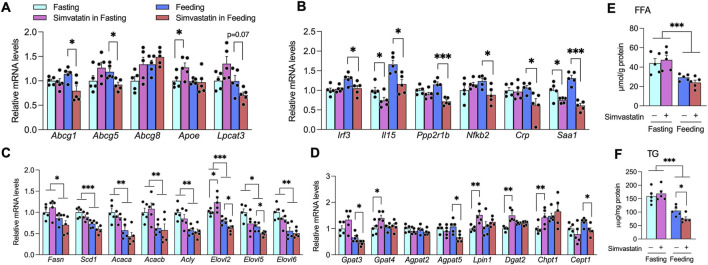
Feeding-phase simvastatin depletes oxysterols and suppresses LXR/SREBP-1c signaling. **(A)** Hepatic expression of canonical LXR targets in A/J mice treated with simvastatin during fasting or feeding. **(B)** Expression of TLR4-dependent NF-κB and IRF3 target genes under the same conditions. **(C)** Hepatic SREBP-1c target genes involved in fatty acid biosynthesis (*Fasn*, *Scd1*, *Acaca*, *Acacb*, *Acly*, *Elovl2*, *Elovl5*, *Elovl6*). **(D)** Additional SREBP-1c targets regulating triglyceride and phosphatidylcholine synthesis. **(E,F)** Hepatic free fatty acid (FFA) **(E)** and triglycerides (TG) **(F)** content in livers of A/J mice treated with simvastatin during fasting versus feeding. Gene expression values were normalized to *Hprt*. Data are mean ± SEM (n = 5 per group). Statistical significance is indicated by *p* < 0.05; *p < 0.01; **p < 0.001 (two-way ANOVA with Tukey’s *post hoc* test).

Oxysterols—such as 24S-, 25-, and 27-hydroxycholesterol—serve as physiological ligands for liver X receptors (LXRα and LXRβ), activating them at concentrations typically found *in vivo* (Fu et al., 2001; Janowski et al., 1996; Lehmann et al., 1997). To determine whether feeding-phase simvastatin—which depletes hepatic oxysterols—alters LXR activity, we first measured the expression of canonical LXR targets that contribute to HDL biogenesis and cholesterol efflux. Notably, *Abcg1*, *Abcg5*, and *Srebf1* (encoding SREBP-1c) were significantly repressed when simvastatin was given with food, but not during fasting (Figures 3C, 6A).

Although activation of *Tlr3*/*Tlr4* by *E. coli*, influenza A, or ligands such as poly I:C (TLR3 agonist) and lipid A (TLR4 agonist) can block LXR signaling in macrophages via IRF-3 competition for the co-activator p300/CBP, we found that simvastatin co-administration with food significantly downregulated IRF-3 and NF-κB targets (*Il15*, *Ppp2r1b*, *Crp*, *Saa1*) (Figure 6B). These results suggest that the IRF-3 pathway is unlikely to mediate the synergistic inactivation of LXR by LPS and simvastatin in the liver, and instead point to oxysterol depletion as the primary mechanism.

Consistent with prior reports that statin exposure can reduce both membrane-bound and nuclear SREBP-1c in an LXR-dependent manner (DeBose-Boyd et al., 2001; Liang et al., 2002; Rong et al., 2017), simvastatin treatment during feeding—but not fasting—led to decreased SREBP-1c expression (Figure 3C). Reduced SREBP-1c transactivation was further evident in the feeding group, where SREBP-1c targets involved in lipogenesis (*Elovl2*, *Elovl5*), triglycerides synthesis (*Gpat3*, *Agpat5*), and phospholipid formation (*Chpt1*, *Cept1*) were suppressed (Figures 6C,D). Quantitatively, feeding-phase simvastatin did not alter hepatic free fatty acid levels but significantly lowered hepatic triglycerides content, consistent with diminished SREBP-1c activity (Figures 6E,F).

Because *de novo* lipogenesis—including phosphatidylcholine synthesis via choline/ethanolamine phosphotransferase 1 (*Cept1*)—is required for full PPARα activation (Guan et al., 2018; Zayed et al., 2021), our mechanistic data support a model in which feeding-driven LPS, together with simvastatin, depletes hepatic oxysterol pools and inactivates LXR. This, in turn, dampens SREBP-1c activity, reduces *de novo* lipogenesis (Figures 6C,D), and limits PPARα activation both during the feeding period and throughout the subsequent daytime fast when HDL-C and glucose were measured (Figure 1D; Supplementary Figure S1). Consequently, mice receiving simvastatin with food display persistently lower fasting blood glucose and HDL-C, in sharp contrast to the HDL-raising, PPARα-activating, and dysglycemic profile observed when simvastatin is administered during the fasting phase.

### 3.7 TLR4 deficiency reverses simvastatin’s effects on HDL cholesterol and glucose metabolism in the feeding state

We observed that LPS suppresses hepatic expression of *CYP7A1*, *CYP27A1*, and *CYP46A1*, thereby priming the liver for further oxysterol depletion by simvastatin during feeding. Previous work has shown that LPS-mediated downregulation of hepatic cytochrome P450 (P450) mRNAs is entirely TLR4-dependent (Chaluvadi et al., 2009). To determine whether TLR4 is also essential for modulating simvastatin’s effects on HDL cholesterol and glucose homeostasis across feeding and fasting states, we used C3H/HeJ mice harboring a spontaneous Tlr4 mutation (*Tlr4* ^*Lps-d*^), which renders them hyporesponsive to LPS (Kamath et al., 2003). These mice, along with wild-type C3H/HeOuJ controls, were fed simvastatin mixed in chow for 5 weeks.

**FIGURE 7:**
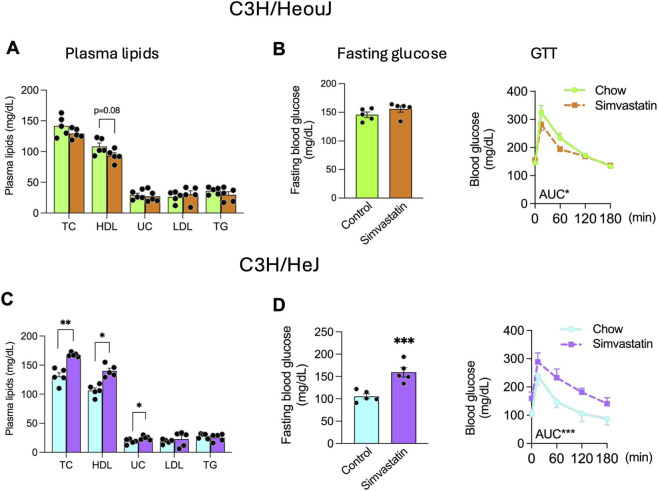
TLR4 deficiency alters simvastatin’s effects on HDL cholesterol and glucose metabolism. **(A,B)** Plasma lipid and glucose measurements in wild-type C3H/HeOuJ mice receiving simvastatin in chow for 5 weeks. **(C,D)** Plasma lipid and glucose measurements in TLR4-deficient C3H/HeJ mice under the same feeding-phase simvastatin regimen. Data are presented as mean ± SEM (n = 5 per group). Statistical significance was assessed by one-way ANOVA: *p* < 0.05; *p < 0.01; **p < 0.001.

In wild-type C3H/HeOuJ mice, simvastatin co-administration with food significantly improved glucose tolerance while modestly lowering HDL cholesterol (p = 0.08), mirroring the results in A/J mice (Figures 1B, 7A,B). In contrast, TLR4-deficient C3H/HeJ mice exhibited significantly elevated HDL cholesterol, total cholesterol, and free cholesterol after simvastatin treatment during feeding (Figure 7C). Moreover, the TLR4-mutant mice developed impaired glucose homeostasis under these conditions, as indicated by elevated fasting blood glucose and glucose intolerance (Figure 7D)—a phenotype that closely resembled A/J mice given simvastatin during fasting (Figure 1D). These findings highlight the critical role of TLR4 in mediating feeding-driven LPS modulation of simvastatin’s regulatory effects on HDL cholesterol and glucose metabolism. Without functional TLR4 signaling, the normal synergy between LPS and simvastatin is disrupted, shifting the metabolic outcomes toward elevated HDL and impaired glucose tolerance.

### 3.8 TLR4 deficiency modifies simvastatin’s influence on LXR/SREBP-1c/PPARα pathways

**FIGURE 8:**
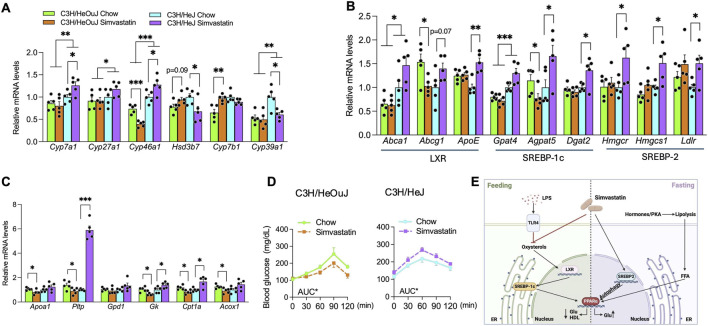
TLR4 deficiency modifies simvastatin’s influence on the LXR/SREBP-1c/PPARα axis. **(A)** Hepatic expression of bile acid/oxysterol-metabolism genes (*Cyp7a1*, *Cyp27a1*, *Cyp46a1*, *Cyp7b1*, *Hsd3b7*) in C3H/HeJ and C3H/HeOuJ mice receiving simvastatin with food. **(B)** Expression of LXR, SREBP-1c, and SREBP-2 target genes in the same livers. **(C)** Expression of *Pparα* and PPARα-regulated genes involved in HDL metabolism and glycerol-driven gluconeogenesis (*Pltp*, *Gk*, *Cpt1a* and others) in C3H/HeJ and C3H/HeOuJ mice. **(D)** Blood glucose levels during GTTs (0, 30, 60, 90, and 120 min) in C3H/HeJ and C3H/HeOuJ mice fed control or simvastatin-enriched chow. **(E)** Schematic summary showing how distinct nutritional states modulate simvastatin’s effects on HDL cholesterol and glucose metabolism via SREBP-2/autophagy/PPARα under fasting, and via LPS–statin convergence on the oxysterol/LXR/SREBP-1c/PPARα pathway under feeding. Data are mean ± SEM (n = 5 per group). Statistical significance is denoted as *p* < 0.05; *p < 0.01; **p < 0.001 (two-way ANOVA with Tukey’s *post hoc* test).

To elucidate how TLR4 modulates simvastatin’s metabolic effects, we compared hepatic expression of key bile acid/oxysterol-metabolizing enzymes between TLR4-deficient (C3H/HeJ) and wild-type (C3H/HeOuJ) mice by quantitative PCR. In wild-type mice, simvastatin treatment suppressed *Cyp46a1* and upregulated *Cyp7b1* (Figure 8A). In contrast, TLR4 deficiency elevated the basal expression of oxysterol-generating enzymes—including *Cyp7a1*, *Cyp27a1*, and *Cyp46a1*. Moreover, in TLR4-deficient livers, simvastatin further increased *Cyp7a1* and *Cyp46a1* expression while downregulating the oxysterol-metabolizing genes *Hsd3b7* and *Cyp39a1* (Figure 8A), suggesting that loss of TLR4 prevents simvastatin-induced depletion of hepatic oxysterols and instead favors their accumulation.

TLR4 deficiency also counteracted simvastatin-mediated inhibition of the LXR/SREBP-1c axis. This was evidenced by increased expression of *Apoe*, *Gpat4*, *Cept1*, and *Cpt1a*, which were otherwise reduced in simvastatin-treated wild-type mice (Figure 8B). These findings suggest that, in the absence of TLR4, simvastatin promotes LXR ligand synthesis and activation rather than suppressing it.

Analysis of the PPARα pathway further showed that simvastatin enhanced PPARα target genes relevant to HDL remodeling and lipid handling (*Pltp*, *Gk*, *Cpt1a*) in TLR4-deficient mice but downregulated these genes in wild-type controls (Figure 8C). To verify that PPARα contributes to dysglycemia in simvastatin-treated TLR4-deficient mice, we conducted glycerol tolerance tests 3 days before completion of the 5-week simvastatin or control treatment (Sumara et al., 2012). Glycerol administration led to higher circulating glucose levels in TLR4-deficient C3H/HeJ mice compared with wild-type C3H/HeOuJ mice, confirming that PPARα-driven, glycerol-fueled gluconeogenesis exacerbates glucose intolerance in TLR4-deficient mice given simvastatin (Figure 8D).

Collectively, these results demonstrate that TLR4 is essential for feeding-driven LPS regulation of simvastatin’s metabolic actions. In TLR4-deficient mice, simvastatin’s usual effects on HDL cholesterol and glucose metabolism are reversed because of disrupted SREBP-1c and PPARα pathways, underscoring the pivotal role of TLR4 in modulating simvastatin’s influence on hepatic lipid and glucose homeostasis.

## 4 Discussion

Statins are well established for lowering LDL-C, modestly raising HDL-C, and reducing ASCVD risk, but they are also associated with a small yet significant increase in the risk of new-onset diabetes (Barter et al., 2010; Cholesterol Treatment Trialists’ Collaboration, 2024). Their efficacy and adverse metabolic effects are tightly linked to endogenous cholesterol metabolism (Ward et al., 2019). Short–half-life statins such as simvastatin are traditionally prescribed in the evening based on the classical concept that hepatic cholesterol synthesis peaks at night. Cholesterol homeostasis, however, reflects the dynamic interplay between hepatic cholesterol synthesis and intestinal absorption of dietary and biliary cholesterol, processes that themselves show diurnal variation and are reciprocally related (Hussain and Pan, 2015; Schroor et al., 2019). More recent work in rodents and humans argues that intrinsic circadian clocks do not directly dictate the cholesterol biosynthetic program; instead, feeding–fasting cycles emerge as the dominant drivers of SREBP-dependent cholesterol biosynthesis, capable of overriding circadian oscillations (Greco et al., 2021; Stokkan et al., 2001; Vollmers et al., 2009). Against this backdrop, our study was designed to dissect how the nutritional state—fasting versus feeding—modulates SREBP-controlled pathways to shape simvastatin action. We show that simvastatin’s effects on HDL-C and glucose metabolism are strongly contingent on whether the drug is administered during fasting or feeding: co-administration with food lowers HDL-C and improves glucose metabolism, whereas administration during fasting modestly raises HDL-C but markedly impairs glucose tolerance. These findings highlight feeding-driven metabolic cues as key determinants of statin responsiveness.

Statins can elevate HDL-C to varying degrees, although the underlying mechanisms remain incompletely defined (Barter et al., 2010). Our data reveal that the fasting-versus feeding-dependent differences in HDL regulation by simvastatin are mediated by PPARα, a nuclear receptor that coordinates HDL metabolism and glycerol-fueled gluconeogenesis. Under fasting conditions, simvastatin robustly induced PPARα and its target genes (e.g., *APOA1* in human hepatocytes and *Pltp* in both human and mouse hepatocytes), which are critical for HDL biogenesis (Bouly et al., 2001; Han et al., 2006; Martin et al., 2001). In parallel, PPARα activation increased expression of glycerol-pathway genes (*Gpd1*, *Gk*, *Aqp9*), providing a mechanistic basis for the observed deterioration in glucose tolerance. In contrast, simvastatin administered with food suppressed PPARα activity and downregulated key targets involved in HDL production and glycerol utilization, indicating that PPARα functions as a metabolic switch that integrates nutritional cues to modulate statin-induced lipid and glycemic responses.

During fasting, FFAs mobilized from adipose tissue supply substrates and ligands for hepatic PPARα (Bideyan et al., 2021). Although synthetic PPARα agonists (e.g., fibrates) strongly induce HDL-related genes, long-chain FFAs alone often have modest or even inhibitory effects (Chakravarthy et al., 2009). Consistent with this, oleic acid by itself did not induce *APOA1* in Huh-7 cells, whereas co-treatment with simvastatin and oleic acid synergistically activated PPARα and markedly enhanced *APOA1* expression. Mechanistically, simvastatin increased SREBP-2 expression and induced autophagy-related genes in fasting liver, and pharmacologic inhibition of autophagy blunted simvastatin-induced *APOA1* upregulation. These findings support a model in which simvastatin promotes SREBP-2–dependent autophagy, facilitating degradation of nuclear receptor corepressors such as NCoR1 and HDAC3 and thereby amplifying FFA-driven PPARα activation to raise HDL-C and stimulate gluconeogenesis (Iershov et al., 2019; Saito et al., 2019) (Figure 8E).

In contrast, when simvastatin was co-administered with food, we observed reduced HDL-C and improved glucose tolerance. We identify feeding-induced LPS as a major determinant of this divergence. Meal ingestion triggers a transient rise in circulating LPS—metabolic endotoxemia—that wanes during fasting (Cani et al., 2007). Our *in vitro* and *in vivo* data indicate that LPS, together with simvastatin, reshapes hepatic oxysterol metabolism by repressing oxysterol-producing enzymes (*Cyp7a1*, *Cyp27a1*, *Cyp46a1*) and upregulating the oxysterol-metabolizing enzyme *Cyp7b1*. The resulting depletion of regulatory oxysterols diminishes LXR activity, downregulates *Srebf1* and its lipogenic targets, and attenuates *de novo* lipogenesis, which is required for full PPARα activation in both postprandial and fasting states (Guan et al., 2018; Zayed et al., 2021). Thus, feeding-driven LPS signaling effectively flips the LXR/SREBP-1c/PPARα axis toward reduced HDL biogenesis and enhanced glycemic control (Figure 8E).

Our experiments using TLR4-deficient C3H/HeJ mice further implicate LPS–TLR4 signaling as a critical gatekeeper of simvastatin’s metabolic actions during feeding. In wild-type C3H/HeOuJ mice, simvastatin with food improved glucose tolerance and modestly lowered HDL-C, mirroring the phenotype in A/J mice. In TLR4-deficient mice, however, the same regimen led to elevated total and HDL-C and worsened glucose tolerance, indicating that intact TLR4 signaling is essential for the canonical feeding-phase response. At the molecular level, loss of TLR4 prevented the feeding-induced suppression of oxysterol-biosynthetic enzymes, preserved LXR activity, and rescued PPARα signaling and its downstream targets (*Pltp*, *Gk*, *Cpt1a*). These findings underscore the central role of TLR4 in linking metabolic endotoxemia to simvastatin-driven remodeling of hepatic lipid and glucose metabolism.

An additional insight from our work is that TLR4 deficiency essentially converts the metabolic response to feeding-phase simvastatin into a fasting-like phenotype, with elevated HDL-C and glycerol-driven hyperglycemia. Glycerol tolerance tests confirmed that PPARα-dependent gluconeogenesis is exaggerated in TLR4-deficient mice given simvastatin, pinpointing TLR4 as a key node coupling LPS signaling, PPARα activation, and systemic glycemic control. Common inbred strains such as C57BL/6J and A/J are widely used to interrogate glucose metabolism, and our previous work has leveraged these backgrounds to dissect sex differences and X-chromosome dosage effects in statin-induced dysglycemia and mitochondrial dysfunction (Zhang et al., 2024), supporting their utility for mechanistic studies of statin-related metabolic phenotypes.

We also acknowledge important limitations of our animal models and how they relate to human pathophysiology. Conventional mice lack CETP and thereby display low LDL-C with HDL-predominant lipoprotein profiles, making them suboptimal for modeling LDL lowering *per se*. To address translational relevance, we relate our findings to prior work in *Apoe* ^−/−^ mice, a well-established hypercholesterolemic model widely used in statin and atherosclerosis research. In these mice, once-daily intragastric simvastatin increased plasma HDL-C, whereas simvastatin admixed in chow reduced HDL-C (Song et al., 2011; Wang et al., 2002)—a fasting-like versus feeding-like contrast that closely parallels the phase-dependent HDL responses we observe in A/J mice. Together, these data support HDL-C and glucose metabolism, rather than LDL-C, as the most informative mechanistic readouts in our study.

Our work has additional limitations that guide future investigations. Although the same nominal simvastatin dose was used in both fasting and feeding protocols, once-daily gavage and continuous dietary intake almost certainly produce different pharmacokinetic profiles. We have therefore initiated a collaborative LC–MS/MS study to quantify simvastatin and its active acid under fasting and feeding conditions and to relate these profiles to 24-h changes in hepatic HMG-CoA reductase activity and cholesterol synthesis markers. These experiments will refine our understanding of dosing-time–dependent statin pharmacokinetics and help bridge our mechanistic mouse data to clinical practice. We also recognize that CLOCK genes and circadian mechanisms likely continue to modulate hepatic lipid and glucose metabolism and may interact with the pathways described here. Nevertheless, our findings indicate that nutritional state is a dominant proximal determinant of simvastatin responses, and future work will be needed to delineate how circadian and feeding cues jointly shape statin chronopharmacology.

In summary, this study demonstrates that feeding and fasting states critically shape simvastatin’s impact on HDL and glucose metabolism via distinct, nutritionally gated pathways. During fasting, simvastatin promotes SREBP-2–driven autophagy and PPARα activation, increasing HDL-C but also enhancing glycerol-fueled gluconeogenesis. During feeding, LPS–TLR4–dependent remodeling of oxysterol metabolism suppresses LXR/SREBP-1c signaling, attenuates *de novo* lipogenesis, and dampens PPARα activity, leading to lower HDL-C and improved glucose tolerance (Figure 8E). These insights provide a mechanistic framework for interindividual variability in statin responses and suggest that aligning statin therapy with nutritional state—and potentially targeting the LPS–TLR4–SREBP/LXR/PPARα axis—may improve lipid-lowering efficacy while mitigating dysglycemic risk.

## Data Availability

The datasets presented in this study can be found in online repositories. The names of the repository/repositories and accession number(s) can be found in the article/Supplementary Material.

## Supplementary material

SUPPLEMENTARY FIGURE S1 Feeding- versus fasting-phase simvastatin dosing differentially alters HDL across the light period Twenty-four–hour plasma lipid profiles in A/J mice treated with simvastatin for 5 weeks either mixed in chow during the active feeding phase (top panels) or by morning oral gavage during the light-phase fasting period (bottom panels). Plasma TC, HDL-C, UC, LDL-C, and TG were measured at 0:00, 6:00, 12:00, and 18:00 (n = 5 per group). Bars represent mean ± SEM; individual mice are shown as dots. Simvastatin given with food consistently shifted HDL-C downward relative to controls, whereas fasting-phase gavage shifted HDL-C upward at corresponding time points. Data are presented as mean ± SEM (n = 5 per group). Statistical significance was determined by one-way ANOVA: **p* < 0.05.

SUPPLEMENTARY FIGURE S2 Simvastatin exerts minimal effects on hepatic core clock gene expression in fasting and feeding states Hepatic mRNA expression of the core clock genes *Bmal1* (*Arntl*) and *Per2* in A/J mice treated for 5 weeks with vehicle or simvastatin (∼16 mg/kg/day). Simvastatin was administered either by oral gavage at ZT04 during the light-phase fasting period (Fasting + Veh, Fasting + Sima) or admixed into chow and consumed during the dark-phase feeding period (Feeding + Veh, Feeding + Sima), as in Figure 1. Livers were collected 5 h after the last gavage dose (ZT09) for the fasting regimen and 5 h after the main feeding bout (ZT05) for the dietary regimen. Gene expression was quantified by qRT-PCR, normalized to *Hprt*, and expressed relative to the fasting-vehicle group. Data are mean ± SEM (n = 5 per group). Statistical significance, where present, is indicated by ***p* < 0.001 (two-way ANOVA with Tukey’s post hoc test).

SUPPLEMENTARY FIGURE S3 PPARα antagonism blunts simvastatin-induced expression of HDL and glycerol-pathway genes in Huh7 cells Huh7 cells were treated with vehicle (Control), simvastatin, the selective PPARα antagonist GW6471, or simvastatin plus GW6471 under the conditions described in Methods. Relative mRNA levels of *APOA1* and *PLTP* (HDL-related genes) and *GPD1* and *GK* (glycerol-pathway genes) were measured by qRT-PCR and normalized to housekeeping genes. Simvastatin alone markedly increased *APOA1*, *PLTP*, *GPD1*, and *GK*, whereas co-treatment with GW6471 largely abolished these transcriptional responses; GW6471 alone had minimal effects. Data are mean ± SEM, with individual data points (black circles) denoting independent biological replicates. Statistical significance is indicated as **p* < 0.01, ***p* < 0.001 (two-way ANOVA with Tukey’s post hoc test).
