# Supplementary material for: TLR4 modulates simvastatin’s impact on HDL cholesterol and glycemic control
Source: Front Pharmacol. 2026 Jan 16;16:1655873. doi: 10.3389/fphar.2025.1655873 (PMC12855043; doi:10.3389/fphar.2025.1655873)
Supplement: Supplementary file 1 [file Table1.docx]

TLR4 Modulates Simvastatin’s Impact on HDL Cholesterol and Glycemic Control

Xiao Tian^1^, Peixiang Zhang ^1*^

^1^ Division of Endocrinology, Diabetes and Nutrition, Department of Medicine, University of Maryland School of Medicine, Baltimore, MD, USA

*** Correspondence:**Corresponding Author
[pzhang@som.umaryland.edu](mailto:pzhang@som.umaryland.edu)

**Table S1: Primers for Quantitative PCR**

| **Name** | **Forward** | **Reverse** |
| --- | --- | --- |
| *Apoa1* | AGACAGCGGCAGAGACTATG | CCCAGAAGTCCCGAGTCAAT |
| *Apoa2* | TGGTCACCATCTGTAGCCTG | AGCTGCTCGTGTGTCTTCTC |
| *Apom* | CTACCAGCGCTTTCTCCTCT | AGTCACTGGTCACTTGCTGG |
| *Abca1* | GCTGCTGTGGAAGAATCTGAC | GTGGCACTCATGTTGTTCGT |
| *Lcat* | CTCCACTTCTTACTGCGGCA | TATGCGCTGCTCCTCTTTCA |
| *Pltp* | TCTGCTGCTGAACATCTCCA | TGGGAGAGCTGTAGACCTGT |
| *Scarb1* | TGCCCCAGGTTCTTCACTAC | TCCTCAAGAAGCGGGGTGTA |
| *Ppara* | GCAGCCTTGAACTTCAGTCC | CATGTTGGATGGATGTGGCC |
| *Cpt1a* | GATGACGGCTATGGTGTTTCC | TTTTGGAATTGGCGGTGAGG |
| *Cpt2* | CATTGGGAAGGAGCTGCATG | GGTCCGGATTGAATGCCATG |
| *Acsl1* | GACCTCTCCATGCAGTCAGT | CTGGTTTGGCTTCCGAGAAC |
| *Acsl5* | CCAGAAAGCCTCACTCGGAA | AAACGTCAGGAGGCAGATCA |
| *Acadm* | TGTGGAGAAGCTGATGAGGG | TTTCCCCTGAAGCAGCAACA |
| *Acadl* | GGAGCATGACATTTTCCGGG | AATGCCGCCATGTTTCTCTG |
| *Acox1* | GAGGGAATTTGGCATCGCAG | ATCTGGAGTTCTTGGGACGG |
| *Gpd1* | GGTGGCTGAGGAGAAGTTCT | AAGCCAAGCCCATCACAGAA |
| *Gk* | CGTGTGAGAAACTTGGACAGC | GACAACGGCAACTGGAACTG |
| *Aqp9* | CTCAAGCAGTCCTCAGTCGA | GGGGAACTTGAACCACTCCA |
| *Pck1* | TGCCTGGATGAAGTTTGATGC | CTCATCGATGCCTTCCCAGT |
| *G6pc1* | AGCTGAACGTCTGTCTGTCC | CAAGCGCGAAACCAAACAAG |
| *Srebf2* | CTTTGTTTCGGCGGCCTTAA | CACTGCTGTTGTTGCCACTG |
| *Hmgcs1* | AAAAGATCCGTGCCCAGTGG | CCAAAGGCTTCCAGTCCACT |
| *Hmgcr* | GTAGGCTGCAAATTGACCCG | TCTTGTTGTTGCCGGTGAAC |
| *Sqle* | TACCGTGTTCTCCAGGAGCT | TGATGAATCGGCCATGGTGA |
| *Lss* | GAATCCTGGGTATTGGGCCT | GTGTTGAGTCCTTCCCAGCT |
| *Sc5d* | AAGAACCAAGTCTCGCGTGA | ACGACGCTAACCATGAGATGA |
| *Dhcr7* | CGAGAGTGCAGAAGAGTGGA | GAAAATGATGCTGGCCAGGG |
| *Dhcr24* | AATGGAAGGAACAGGGCAGT | ATAGACACCAAGGGCTCCAC |
| *Ldlr* | CCAGTGTGACCGTGAACATG | CACTCGTTGGTCTTGCACTC |
| *Atg7* | TGTACAGCCTGTTCACCCAA | GCTTCAGACAGTCTCCTCGT |
| *Atg12* | TTAAACTGGTGGCCTCGGAA | CATTTCTGGCTCATCCCCATG |
| *Becn1* | AGCTGGAGTTGGATGACGAA | TGATTGTGCCAAACTGTCCG |
| *Cyp7a1* | GCCCTGAAGCAATGAAAGCA | TCCTCCTTAGCTGTCCGGAT |
| *Cyp27a1* | GATGAGACAGGAGGGCAAGT | TAAGGCATCCGTGTAGAGCG |
| *Cyp46a1* | ACCTCAGTCATTGTCACGAGT | CCCCAAACACAGTCTGAAGC |
| *Hsd3b7* | GCGCTTTGGAGGTCGTCTAT | GCAGAAATACACCTGGCCAC |
| *Cyp7b1* | TTCTCTTTGCCGCCACCTTA | CCATGCCAAGATAAGGAAGCC |
| *Cyp39a1* | ACTTTACTGGGCCGCTAACT | AATGACCTGCAGATCCGTGA |
| *Abcg1* | CGGCCGTGAACATCGAATTC | TCTCCCTGTATCCTGCCAGA |
| *Abcg5* | TGTACATCGAGAGTGGCCAG | GCAGCCATTCACAAACACCT |
| *Abcg8* | GACAGCTTGTTCTCCTCGGA | GCCTGTGATCACGTCGAGTA |
| *Apoe* | AAGATGGGGTTCTCTGGGTG | ACTCGAGCTGATCTGTCACC |
| *Lpcat3* | GCGGCTCATCTTCTCCATCT | TAAGCAATTGAGAGGCCCGT |
| *SREBP-1c* | GGAGCCATGGATTGCACATT | GGCCCGGGAAGTCACTGT |
| *Irf3* | TCGGAGGCTTAGCTGACAAA | AGGGCAAAATCCGCGGTTT |
| *Il15* | CCTTGCTGGTGAGGTCCTTA | TGCCCAGGTAAGAGCTTCAAT |
| *Ppp2r1b* | TCGGGGTAGAAAGGACTCGA | CCACCGTAGCCAAACTTTCC |
| *Nfkb2* | GGAAGGGCTCGGAAAGAAGT | GGCCTGGATCGTAGCAATTG |
| *Crp* | AGCTACTCTGGTGCCTTCTG | GGCTTCTTTGACTCTGCTTCC |
| *Saa1* | ATCACCAGATCTGCCCAGGA | TCATGTCAGTGTAGGCTCGC |
| *Fasn* | ATGGCCGCGGTTTAAATAGC | ACATACCGGCTATCACCACC |
| *Scd1* | TACTGGTTCCCTCCTGCAAG | GCCGTGCCTTGTAAGTTCTG |
| *Acaca* | ATGTCTGGCTTGCACCTAGT | AAGACCACCGACGGATAGATC |
| *Acly* | CTAAAACCTCGCCTGGGACA | TGCACACGTAGAACTCCTCC |
| *Elovl2* | TTCCCACCTTCATCCTCACC | TGTAACCTCCTTCCCAGCTG |
| *Elovl5* | ACTCACCCTGCTGTCTCTCT | AGAGGACGCGGATGATCTTC |
| *Elovl6* | ATCTTTGGTGGTCGGCATCT | GCATGTAAGCACCAGTTCGAA |
| *Gpat3* | TCCTTTTACCCTCGGCCTTC | CGAAGACCAGAGAGCCCTTT |
| *Gpat4* | TCATCGTCCCTGCCATCTTT | ATTCCGTTGGTGTAGGGCTT |
| *Agpat2* | TACGCCAAGGTCGGTCTCTA | TTGAAGGACCGAACGAACCA |
| *Agpat5* | ACACCGGGGTCCAGATATTG | CAGTACGTAGCGCACATGTC |
| *Lpin1* | CGGCCTGCTGATGTGTATTC | AGGGGAAGTCACAGGAATGG |
| *Dgat2* | CTTCTCTGTCACCTGGCTCA | TGTTCCAGTCAAATGCCAGC |
| *Chpt1* | CCCAACACCATCACCCTCAT | TCCTTCTGGCTTGTTTCCCA |
| *Cept1* | TGCCCCAAATCTCATCACCA | TTCTTCTGGCCTGTTTCCCA |
| *Hprt* | AGTCCCAGCGTCGTGATTAG | ACACAGAGGGCCACAATGT |
| *Bmal1* | AGCCCGCTGAACATCACAAGT | AGCCTGCCCTGGTAATAGTCCA |
| *Per2* | ACCTTGAAGTATGCCCTGCGGA | AGAAACCAGGGACACAGCCACA |
| *B2m* | CTGACCGGCCTGTATGCTAT | GGATTTCAATGTGAGGCGGG |
| *APOA1* | CTCAAAGACAGCGGCAGAGA | TGTCCCAGTTGTCAAGGAGC |
| *PLTP* | CACCTACTTTGGGAGCATTGT | TGGTGACGCTAGCAGTGAC |
| *GPD1* | CAGCATCCTCCAGCACAAGG | TCTGGATGATTCTGCAGGCA |
| *GK* | TGTGAGAAACTTGGACAGCTCA | ATCAAGCCACACCACAGCAT |
| *CYP46A1* | CTACGAGCACATCCCCGGG | CCGCACAACAGGTCCATACT |
| *HPRT1* | CAGACTTTGCTTTCCTTGGTCA | TTCGTGGGGTCCTTT TCACC |
| *B2M* | AGATGAGTATGCCTGCCGTG | CATCCAATCCAAATGCGGCA |
