# Supplementary material for: TLR4 modulates simvastatin’s impact on HDL cholesterol and glycemic control
Source: Front Pharmacol. 2026 Jan 16;16:1655873. doi: 10.3389/fphar.2025.1655873 (PMC12855043; doi:10.3389/fphar.2025.1655873)
Supplement: Supplementary file 2 [file Presentation1.pptx]

## Slide 1
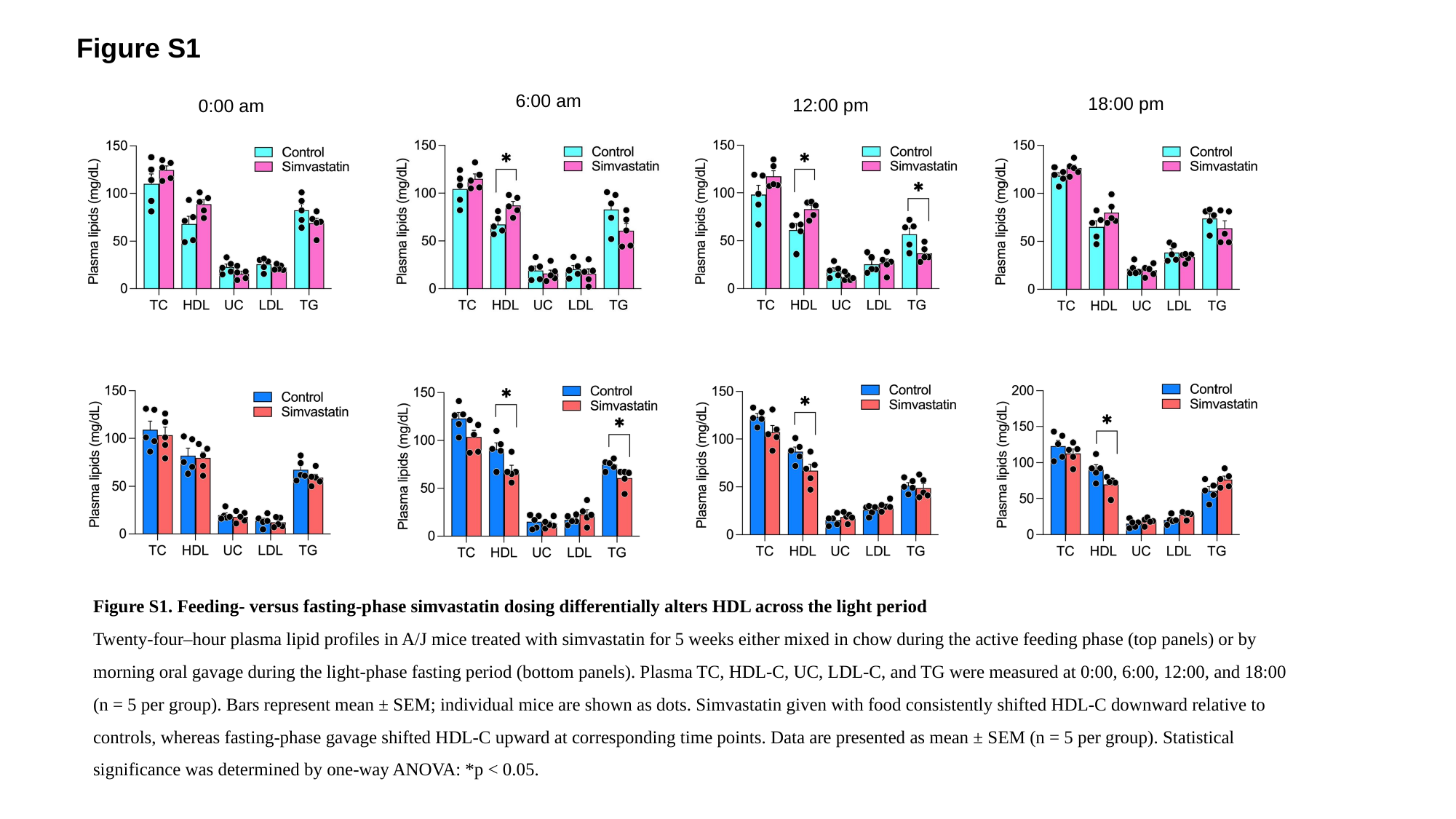

Figure S1
6:00 am
18:00 pm
12:00 pm
0:00 am
Figure S1. Feeding- versus fasting-phase simvastatin dosing differentially alters HDL across the light period
Twenty-four–hour plasma lipid profiles in A/J mice treated with simvastatin for 5 weeks either mixed in chow during the active feeding phase (top panels) or by morning oral gavage during the light-phase fasting period (bottom panels). Plasma TC, HDL-C, UC, LDL-C, and TG were measured at 0:00, 6:00, 12:00, and 18:00 (n = 5 per group). Bars represent mean ± SEM; individual mice are shown as dots. Simvastatin given with food consistently shifted HDL-C downward relative to controls, whereas fasting-phase gavage shifted HDL-C upward at corresponding time points. Data are presented as mean ± SEM (n = 5 per group). Statistical significance was determined by one-way ANOVA: *p < 0.05.

## Slide 2
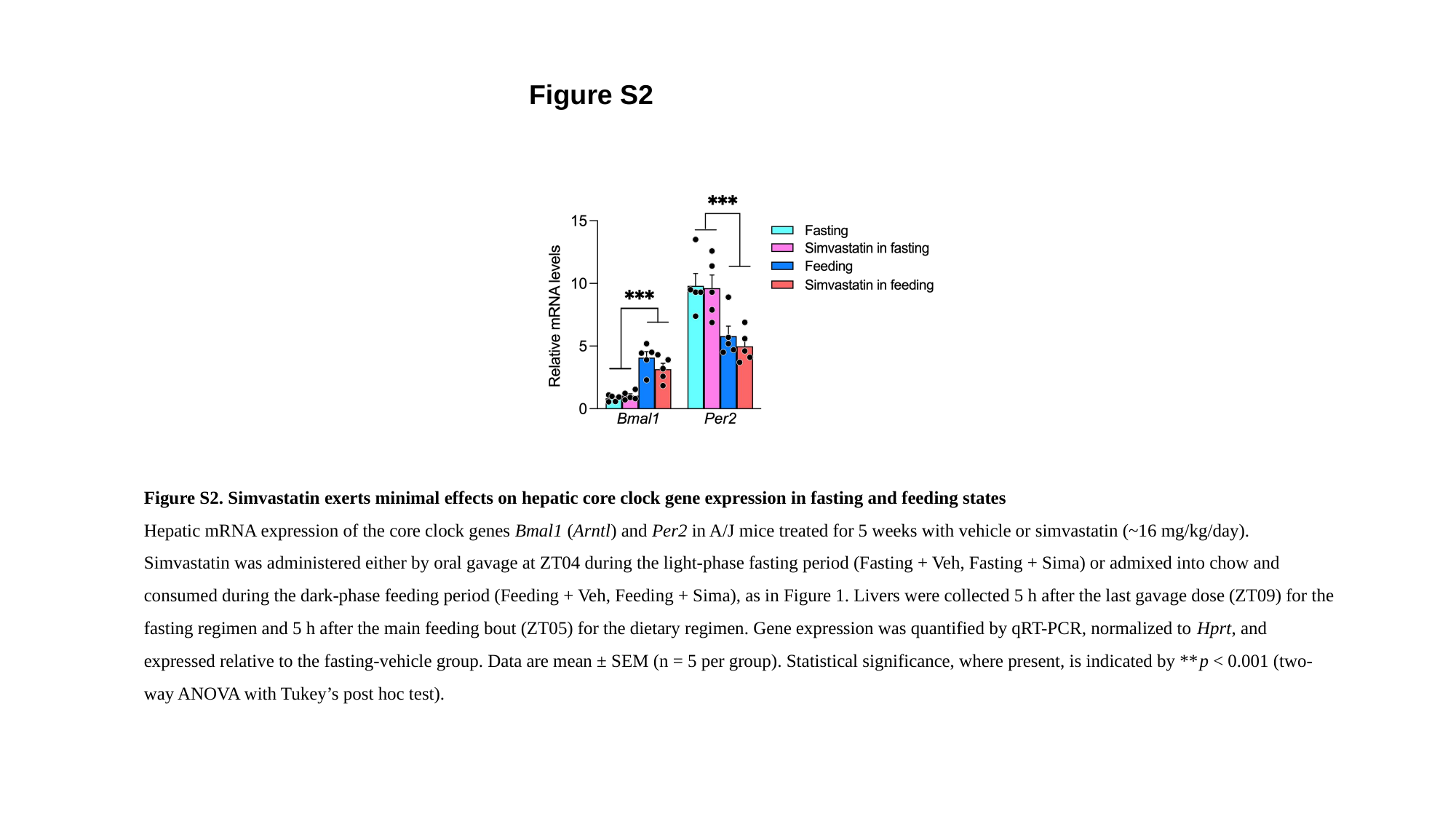

Figure S2
Figure S2. Simvastatin exerts minimal effects on hepatic core clock gene expression in fasting and feeding states
Hepatic mRNA expression of the core clock genes Bmal1 (Arntl) and Per2 in A/J mice treated for 5 weeks with vehicle or simvastatin (~16 mg/kg/day). Simvastatin was administered either by oral gavage at ZT04 during the light-phase fasting period (Fasting + Veh, Fasting + Sima) or admixed into chow and consumed during the dark-phase feeding period (Feeding + Veh, Feeding + Sima), as in Figure 1. Livers were collected 5 h after the last gavage dose (ZT09) for the fasting regimen and 5 h after the main feeding bout (ZT05) for the dietary regimen. Gene expression was quantified by qRT-PCR, normalized to Hprt, and expressed relative to the fasting-vehicle group. Data are mean ± SEM (n = 5 per group). Statistical significance, where present, is indicated by **p < 0.001 (two-way ANOVA with Tukey’s post hoc test).

## Slide 3
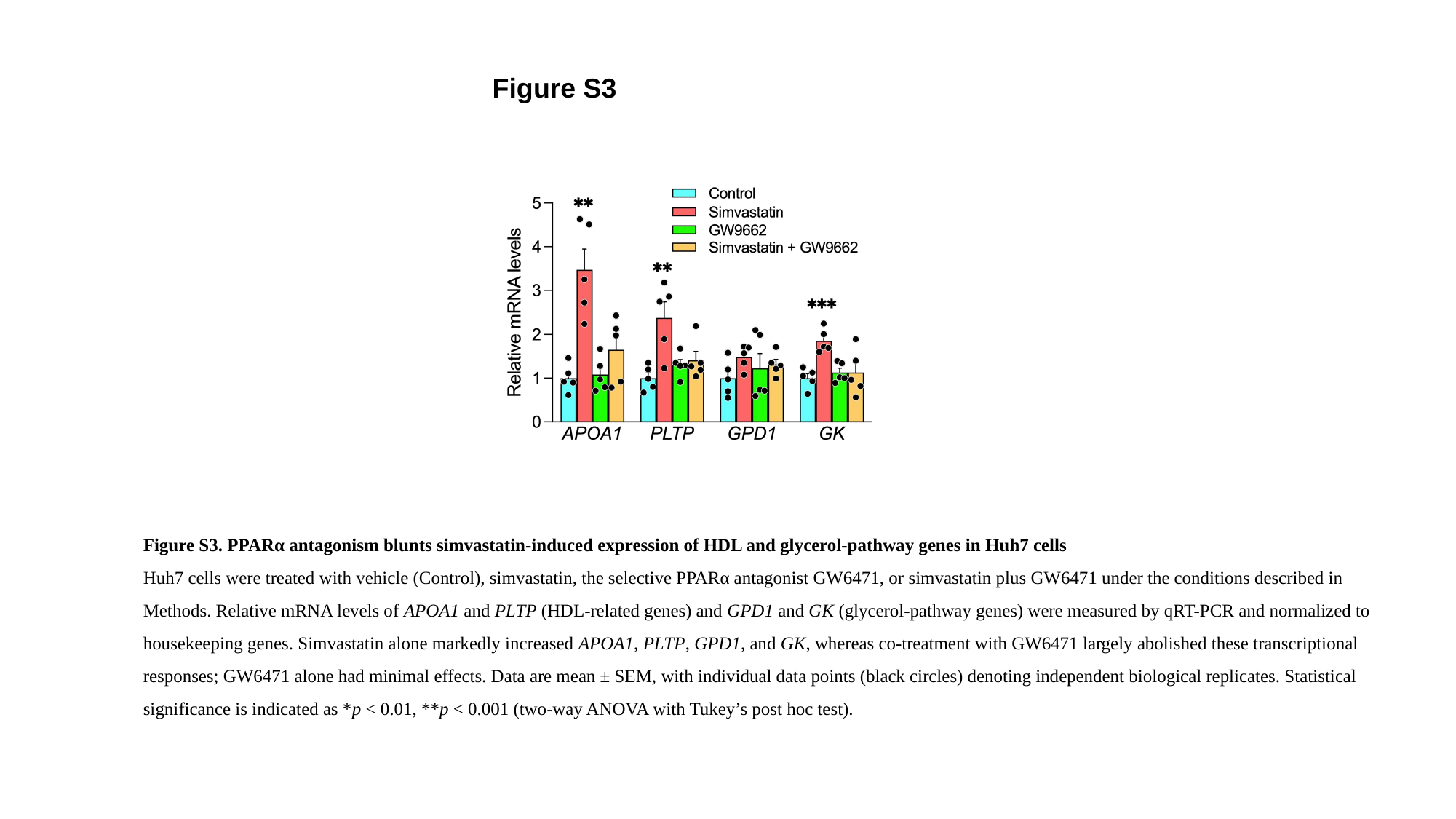

Figure S3
Figure S3. PPARα antagonism blunts simvastatin-induced expression of HDL and glycerol-pathway genes in Huh7 cells
Huh7 cells were treated with vehicle (Control), simvastatin, the selective PPARα antagonist GW6471, or simvastatin plus GW6471 under the conditions described in Methods. Relative mRNA levels of APOA1 and PLTP (HDL-related genes) and GPD1 and GK (glycerol-pathway genes) were measured by qRT-PCR and normalized to housekeeping genes. Simvastatin alone markedly increased APOA1, PLTP, GPD1, and GK, whereas co-treatment with GW6471 largely abolished these transcriptional responses; GW6471 alone had minimal effects. Data are mean ± SEM, with individual data points (black circles) denoting independent biological replicates. Statistical significance is indicated as *p < 0.01, **p < 0.001 (two-way ANOVA with Tukey’s post hoc test).
